# Muscle magnetic resonance imaging in congenital myasthenic syndromes

**DOI:** 10.1002/mus.25035

**Published:** 2016-02-22

**Authors:** Sarah Finlayson, Jasper M. Morrow, Pedro M. Rodriguez Cruz, Christopher D.J. Sinclair, Arne Fischmann, John S. Thornton, Steve Knight, Ray Norbury, Mel White, Michal Al‐hajjar, Nicola Carboni, Sandeep Jayawant, Stephanie A. Robb, Tarek A. Yousry, David Beeson, Jacqueline Palace

**Affiliations:** ^1^ Nuffield Department of Clinical NeurosciencesUniversity of Oxford and Oxford Radcliffe Hospitals NHS TrustOxfordUK; ^2^ MRC Centre for Neuromuscular Diseases, UCL Institute of NeurologyLondonUK; ^3^ University of Oxford Centre for Clinical Magnetic Resonance ResearchJohn Radcliffe HospitalOxfordUK; ^4^ Department of PaediatricsUniversity of Oxford and Children's HospitalOxfordUK; ^5^ Neurology DepartmentHospital San Francesco of NuoroSardiniaItaly; ^6^ Dubowitz Neuromuscular Centre, Institute of Child Health and Great Ormond Street HospitalLondonUK; ^7^ Neurosciences Group, Weatherall Institute of Molecular Medicine, Nuffield Department of Clinical Neurosciences, University of OxfordOxfordUK

**Keywords:** congenital myasthenia, diagnosis, genetic, imaging, MRI, muscle

## Abstract

**Introduction:**

In this study we investigated muscle magnetic resonance imaging in congenital myasthenic syndromes (CMS).

**Methods:**

Twenty‐six patients with 9 CMS subtypes and 10 controls were imaged. T1‐weighted (T1w) and short‐tau inversion recovery (STIR) 3‐Tesla MRI images obtained at thigh and calf levels were scored for severity.

**Results:**

Overall mean the T1w score was increased in *GFPT1* and *DPAGT1* CMS. T1w scans of the AChR‐deficiency, *COLQ*, and *CHAT* subjects were indistinguishable from controls. STIR images from CMS patients did not differ significantly from those of controls. Mean T1w score correlated with age in the CMS cohort.

**Conclusions:**

MRI appearances ranged from normal to marked abnormality. T1w images seem to be especially abnormal in some CMS caused by mutations of proteins involved in the glycosylation pathway. A non‐selective pattern of fat infiltration or a normal‐appearing scan in the setting of significant clinical weakness should suggest CMS as a potential diagnosis. Muscle MRI could play a role in differentiating CMS subtypes. *Muscle Nerve* **54**: 211–219, 2016

**Abbreviations**
AChR: acetylcholine receptor
AIMG: autoimmune myasthenia gravis
CMS: congenital myasthenic syndrome
MGADL: myasthenia gravis activities of daily living
MRC: Medical Research Council
QMG: quantitative myasthenia gravis
SCS: slow channel syndrome
STIR: short‐tau inversion recovery
T1w: T1‐weighted
T2w: T2‐weighted

Congenital myasthenic syndromes (CMS) are a group of inherited disorders that result from gene mutations affecting neuromuscular junction structure and function.^1^, ^2^, ^3^ They are characterized by fatigable muscle weakness, and the majority are autosomal recessive. CMS are treatable disorders, and the choice of treatment is governed by subtype. Certain drugs used to treat some forms of CMS can cause worsening in others, underscoring the importance of establishing a specific genetic diagnosis. Currently, at least 21 genes have been implicated in causing CMS, and hundreds of different mutations have been reported. Therefore, genetic screening can be time consuming. Although the pattern of muscle weakness usually varies according to the specific mutation, the phenotypes can sometimes be difficult to distinguish from other forms of CMS, seronegative autoimmune myasthenia gravis (AIMG), and congenital myopathies.

Muscle magnetic resonance imaging has played an increasing role in the diagnosis of many muscle disorders.^4^, ^5^, ^6^ It is non‐invasive, quick, and generally well tolerated. Importantly, it can identify distinct patterns of muscle involvement, which may help target genetic analysis and those muscles most suitable for biopsy.^7^ Most studies have concentrated on lower limb imaging, where most clinical experience lies. However, whole‐body MRI, or MRI of the truncal and upper limb muscles, is beginning to be explored.^5^, ^8^, ^9^, ^10^ The majority of findings described relate to hyperintensity on T1‐weighted (T1w) images, reflecting fatty infiltration of muscle. Another finding is hyperintensity on fat‐suppressed T2‐weighted (T2w) sequences, such as short‐tau inversion recovery (STIR), likely reflecting increased water content due to inflammation or increased blood flow.^11^ It is postulated that STIR imaging may reveal earlier changes than T1w imaging, before fatty infiltration has had a chance to develop or become evident on imaging.^4^

Analysis in published reports is often undertaken using semiquantitative descriptive rating scales of T1w findings.^12^, ^13^, ^14^, ^15^, ^16^ The main focus has been on identifying patterns of muscle involvement, which can differ strikingly between conditions, and can help direct genetic testing in inherited myopathies and dystrophies.^5^ Retrospective findings of muscle MRI in 6 children with *DOK7* CMS have been reported in brief.^17^ MRI was abnormal in 2 of the 6 children, with mild, non‐specific, and non‐selective atrophic changes. The finding of a relatively normal muscle MRI in a child who is markedly weak has been suggested to possibly indicate a myasthenic syndrome.^18^ However, this has not been confirmed by reported data, and no analysis of MRI that compares findings in different CMS subtypes has been reported.

Although many CMS subtypes have been associated with mild myopathic features on muscle biopsy,^19^ some subtypes have specific pathophysiological features suggesting likely greater secondary muscle damage. Slow channel syndrome (SCS)^20^ and *COLQ* CMS^21^ are associated with an excitotoxic end‐plate myopathy with degeneration of postsynaptic junctional folds. Similar degeneration and other myopathic features have been described in *DOK7* CMS,^22^ which can mimic a limb‐girdle myopathy. The glycosylation pathway subtypes, including *GFPT1*,^23^ *DPAGT1*,^24^ and *ALG2* CMS,^25^ are associated with tubular aggregates in muscle fibers, which are believed to represent aggregations of misfolded proteins.^26^ Therefore, it is possible that some CMS patients may exhibit abnormal muscle MRI, and there may be diagnostically useful differences between subtypes. Any differences identified may help to direct genetic testing, thus enabling early and appropriate symptomatic treatment.

The aims of this study were to define the nature and extent of T1w and STIR abnormality on calf‐ and thigh‐level MRI in CMS patients, to examine whether diagnostically useful differences between subtypes exist, and to identify any correlation with clinical severity measures.

## METHODS

### Recruitment of Participants and Ethics Approval

This prospective imaging study was granted ethics approval by South Central Research Ethics Committee A (11/SC/0157). Informed consent for use of data was obtained (OXREC B: 04.OXB.017 and Oxfordshire REC C 09/H0606/74).

Patients with genetically confirmed congenital myasthenic syndromes were recruited via the nationally commissioned CMS service based in Oxford. All subtypes of CMS were included. Thus, all patients available with the rarer syndromes (glycosylation pathway mutations, *COLQ*, SCS, *CHAT*) and sequential clinic patients with the common CMS subtypes [*RAPSN*, acetylcholine receptor (AChR)–deficiency syndrome and *DOK7*], ≥ 6 years of age and without a history of another neuromuscular disorder, were invited to participate. Approximate age‐ and gender‐matched healthy controls were also recruited.

In addition, 3‐T MRI images of a patient with *ALG2* CMS, whose scans were performed at another center using a different imaging protocol, were also reviewed. These findings were not incorporated in the blinded data and are discussed separately.

### Clinical Parameters

Patient clinical data were collected including genetic subtype. Concurrent clinical assessments included quantitative myasthenia gravis (QMG) score, timed 10‐meter walk, myasthenia gravis activities of daily living (MGADL) score, and Medical Research Council (MRC) score of lower limb muscle strength. The QMG score was initially developed for evaluation of therapy for AIMG and is based on quantitative testing of sentinel muscle groups.^27^ The measure comprises 13 parameters, each scored from 0 to 3. Patients can score up to a maximum of 39 points, and a higher score reflects greater weakness. The QMG score has also been used for the assessment of CMS.^28^, ^29^ The MGADL score is an 8‐question survey designed to grade clinical severity in AIMG where it has been shown to correlate with the QMG score.^30^ The mean of right and left leg raise times, which we have found clinically useful to monitor response to treatment, was extracted from the QMG score.

### MRI Sequences

Subjects were scanned at 3 T (TIM Trio, Siemens, Erlangen, Germany) lying feet first in the supine position with a spine matrix, and 2 surface array coils, including a peripheral angiography coil to receive the signal from the thigh and calf levels of both limbs. The MRI protocol was based on a clinical imaging protocol from the MRC Centre for Neuromuscular Diseases at the National Hospital for Neurology and Neurosurgery, London, UK.^31^ This comprised T1w axial imaging [typical parameters: repetition time (TR)/echo time (TE) = 575/9.7 ms] and axial STIR imaging [typical parameters TR/TE/inversion time (TI) = 5,200/38/220 ms], both with 4‐mm slice thickness and 2‐mm slice gap. Slice center positioning was midway between the greater trochanter of the femur and the patella in thigh‐level sequences and midway between the patella and the talus in calf‐level sequences. Overall scanning time was usually 30 minutes or less and did not require use of sedation.

### MRI Analysis

Patient and control scans were intermixed and analyzed by 2 experienced assessors blinded to clinical details (J.M. and T.A.Y.). The following muscles were analyzed bilaterally: rectus femoris; vastus lateralis; vastus intermedius; vastus medialis; biceps femoris long head; biceps femoris short head; semitendinosus; semimembranosus; adductor magnus; adductor longus; sartorius; gracilis; tibialis anterior; extensor hallucis longus; peroneus longus; lateral gastrocnemius; medial gastrocnemius; soleus; and tibialis posterior.

These 38 muscles were assessed on T1w sequences for the presence of fatty infiltration using the semiquantitative Mercuri score (Table 1).^32^

**Table 1:** Mercuri grades for T1w images

0	Normal appearance.
1	Early “moth‐eaten” appearance, with scattered small areas of increased signal.
2a	Late “moth‐eaten” appearance, with numerous discrete areas of increased signal with beginning confluence, comprising less than 30% of the volume of the individual muscle.
2b	Late “moth‐eaten” appearance, with numerous discrete areas of increased signal with beginning confluence, comprising 30%–60% of the volume of the individual muscle.
3	“Washed‐out, fuzzy” appearance due to confluent areas of increased signal.
4	“End‐stage” appearance, muscle replaced by increased density connective tissue and fat, with only a rim of fascia and neurovascular structures distinguishable.

The same muscles were assessed on STIR sequences and were graded on a previously described 3‐point scale as follows: 0 = normal; 1 = mild; or 2 = marked hyperintensity.^31^ The T1w (Mercuri) and STIR scoring data were analyzed for any pattern of selective muscle involvement.

### Categorizing Severity of Muscle Involvement for each Patient

An overall categorization of both T1w and STIR sequences was developed based on the findings in the healthy volunteer scans (Table 2). Categorizations were determined for thigh‐ and calf‐level muscles for each of the 2 sequences of each subject.

**Table 2:** Severity categorization of T1w and STIR scores

Categorization	Symbol	T1w results	STIR results
Normal	−	All muscles Mercuri grade 0–1	All muscles normal intensity
Mild limited	+/−	≤50% muscles Mercuri grade 2a	Mild hyperintensity in ≤ 25% of muscles
Mild extensive	+	>50% muscles Mercuri grade 2a	Mild hyperintensity in > 25% of muscles
Marked	++	Any muscles Mercuri grade 2b or more	Any muscles with marked hyperintensity

T1w, T1‐weighted; STIR; short‐tau inversion recovery.

In addition, a mean T1w score was established for each patient to compare overall fatty infiltration between subjects. To calculate this, Mercuri scores for each muscle were first modified to contain only numerical values as follows: grade 0 = 0; grade 1 = 1; grade 2a = 2; grade 2b = 3; grade 3 = 4; and grade 4 = 5. The mean was then calculated by dividing the sum of modified Mercuri scores by the total number of muscles analyzed.

### Statistical Analysis

Statistical analyses were performed using GraphPad Prism version 6 (GraphPad Software, La Jolla, California) or SPSS version 22.0 (IBM SPSS, Armonk, New York). The Spearman rank correlation coefficient (rho) was used to assess the correlation between mean T1w score and clinical parameters. Individual modified Mercuri score data from all thigh and calf muscles of the entire CMS group, and the mean T1w scores of each of the different subtypes were compared with the control group using the Mann–Whitney *U*‐test. It is important to note that the small group sizes, inevitable due to the rarity of CMS, limits meaningful additional statistical analysis, and therefore much of the analysis is descriptive.

## RESULTS

### Subject Demographics and Clinical Parameters

A total of 26 subjects (14 women) with 9 CMS subtypes were recruited, including: AChR‐deficiency syndrome, 4; *RAPSN*, 3; *DOK7*, 5; SCS, 5; *COLQ*, 3; *CHAT*, 1; *GFPT1*, 2; *DPAGT1*, 2; *ALG14*, 1; and 10 healthy controls (6 women). Mean age was 38.4 (range 13–70) years in the CMS cohort and 35.7 (range 18–54) years in the control group. Scanning was incomplete in 4 because of technical issues or inability to tolerate lying flat: subject 10 (*DOK7*) only had thigh imaging; subject 15 (SCS) only had calf imaging; subject 13 (SCS) had the right thigh and both calves imaged; and control subject 9 did not have calf STIR imaging. CMS subtype mean MGADL and QMG scores are shown in Table 3. Detailed clinical parameters for individual subjects are shown in Table S1 (refer to Supplementary Material, available online).

**Table 3:** Clinical and imaging findings by CMS subtype

CMS subtype	Subjects by gender (*N*)	Current age, years (range)	MGADL score	QMG score	T1w severity scores (*N*)		Mean T1w score
					Thigh	Calf	
AChR def	2M:2W	24 (16–34)	8.3 (6–9)	17.5 (13–24)	— (1), +/− (3)	— (1), + /− (3)	0.80 (0.26–1.29)
*RAPSN*	2M:1W	51.3 (41–63)	3.7 (2–5)	9 (6–14)	— (1), +/− (1), + + (1)	+/− (1), + (1), + + (1)	1.30 (0.89–1.76)
*DOK7*	2M:3W	45.8 (25–70)	6.6 (0–11)	13.2 (3–16)	— (2), +/− (2), + (1)	+/− (3), + (1), ND (1)	1.15 (0.74–1.74)
SCS	3M:2W	45 (24–66)	6.2 (3–9)	12.4 (3–22)	— (1), +/− (2), + + (1), ND (1)	+/− (2), + (1), + + (2)	1.66 (0.89–3.15)
*COLQ*	3W	15.7 (13–18)	4.3 (1–6)	14.7 (7–19)	— (2), +/− (1)	— (2), + /− (1)	0.55 (0.11–1.00)
*CHAT*	1M	16	6	13	— (1)	+/− (1)	0.53
*GFPT1*	2M	32 (25–39)	2.5 (2–3)	10 (7–13)	+ + (2)	+ + (2)	2.51 (2.29–2.74)
*DPAGT1*	2W	57.5 (57–58)	5.5 (3–8)	18	+ + (2)	+ (1), + + (1)	2.47 (1.92–3.03)
*ALG14*	1W	52	5	15	+/− (1)	+/− (1)	1.21

Group means for each subtype are shown (range in parentheses) for current age, MGADL, QMG, and mean T1w score. AChR def, acetylcholine receptor‐deficiency syndrome; SCS, slow channel syndrome; N, number; W, woman; M, man; QMG, quantitative myasthenia gravis; MGADL, myasthenia gravis activities of daily living; T1w, T1‐weighted; STIR, short‐tau inversion recovery; ND, not done; —, normal; + /−, mild limited changes; + , mild extensive changes; + +, marked changes (as defined in the methods). The number of subjects with each severity score is shown in parentheses.

### T1w Sequences

Overall, in CMS we found a non‐selective distribution of fat infiltration, or a scan within normal limits (particularly in those < 20 years) in the setting of significant clinical weakness.

### CMS Compared with Controls

**Figure 1:**
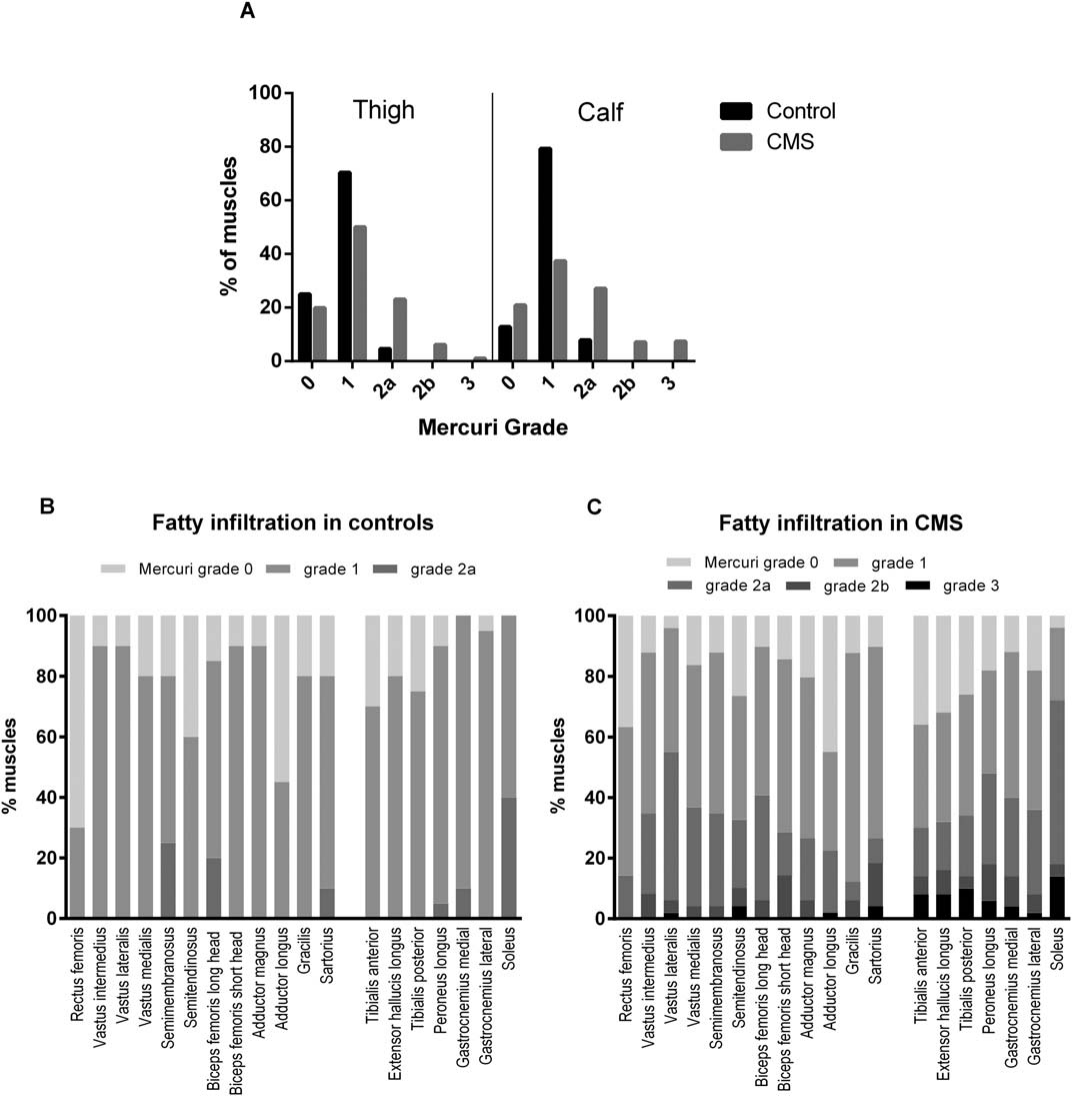
Fatty infiltration of muscles. **(A)** Proportion of muscles by Mercuri grade in the congenital myasthenic syndrome and control cohorts. Thigh and calf data are shown separately. **(B)** The range of Mercuri scores in each muscle analyzed in the control group and **(C)** in patients with CMS. Although higher Mercuri grades are more frequent in the muscles of CMS patients compared with the control group, all muscles are affected similarly.

The Mercuri scores, which indicate the degree of fatty infiltration, were greater in the CMS group (thigh muscles, *P* < 0.0001; calf muscles, *P* < 0.0001). Mercuri grade 0 or grades 1 were found in 229 of 240 (95.4%) control thigh and 129 of 140 (92.1%) control calf muscles compared with 411 of 588 (69.9%) CMS thigh and 204 of 350 (58.3%) CMS calf muscles (Fig. 1A). Grades 2b or 3 were only seen in CMS muscles. No muscles were scored grade 4.

Severity scores in CMS patients (Table 3) and controls (Table 4) showed normal or mild limited changes in 10 of 10 control patient thighs and calves, compared with only 18 of 25 CMS patient thighs and 15 of 25 CMS patient calves. Greater severity scores were only seen in CMS patients; there were mild extensive changes in 1 of 25 thighs and 4 of 25 calves, and marked changes in 6 of 25 thighs and 6 of 25 calves.

**Table 4:** Imaging findings in control subjects

Control ID	Age (years)	Thigh		Calf		Mean T1w score
		T1w	STIR	T1w	STIR	
1	18	—	—	—	—	0.95
2	41	+/–	—	+/–	—	1.05
3	23	+/–	—	+/–	+/–	1
4	31	—	—	—	+	0.53
5	54	+/–	—	+/–	+/–	1.29
6	54	—	—	+/–	+/–	0.97
7	26	—	—	—	+/–	0.16
8	49	—	—	—	—	0.92
9	31	—	—	—	ND	0.66
10	30	—	—	—	+/–	1.00
Mean	35.7					0.85

T1w, T1‐weighted; STIR, short‐tau inversion recovery, ND, not done; —, normal; + /−, mild limited changes; + , mild extensive changes; + +, marked changes.

The mean overall T1w score in controls was 0.85 (range 0.16–1.29), and in CMS was 1.33 (range 0.11–3.15) (*P* = not significant).

### Comparison of Different CMS Subtypes

When present, a non‐selective pattern of muscle involvement was identified in all CMS subtypes (Fig. 1B and C). The numbers were too small within the subtype groups to reliably determine significant differences.

The following CMS subtypes had at least 1 subject with mild extensive or marked fatty infiltration: *RAPSN*; *DOK7*; SCS; *GFPT1*; and *DPAGT1*. However, in SCS and in *RAPSN* and *DOK7* CMS, a wide range of severity was observed, with some patients from each group inhabiting the same severity categories as normal controls. Patients with *GFPT1* and *DPAGT1* subtypes had the most severe fatty infiltration; all 4 subjects with mutations in either of these genes were categorized as having marked or mild extensive changes on both thigh and calf imaging.

**Figure 2:**
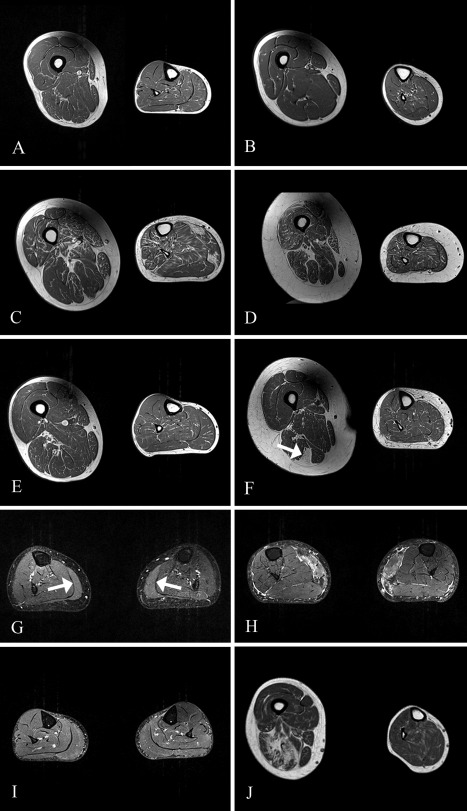
T1w and STIR images. Axial thigh (left) and calf (right) T1w images are shown **(A)**–**(F)** and **(J)**. On T1w images, muscle has a dark gray appearance, and fat and bone appear white. **(A)** The healthy control subject with the highest mean T1w score. **(B)** AChR deficiency, **(C)** *GFPT1*, **(D)** SCS, **(E)** *DOK7*, and **(F)** *RAPSN* CMS. Note the focal areas of muscle with the same signal intensity as subcutaneous fat in semitendinosus in **(F)** (arrow). Bilateral calf STIR imaging is shown in **(G)**–**(I)**. On STIR images the fat signal is suppressed, and white indicates other tissue types with high water content, including blood vessels and areas of “edema” within muscles. **(G)** Mild hyperintensity is seen bilaterally in distal medial gastrocnemius (arrows) in a subject with *CHAT* mutations. **(H)**. Marked STIR hyperintensity in medial gastrocnemius bilaterally in a subject with *GFPT1* mutations. **(I)** A “central stripe” can be seen bilaterally in medial gastrocnemius of this healthy control. **(J)** Three‐Tesla T1w MRI scans from a patient with *ALG2* congenital myasthenic syndrome. Note the predominant fatty infiltration in thigh rather than calf musculature.

Mean T1w scores varied by CMS subtype (Table 3), which again demonstrated the most abnormal scores in the *GFPT1* and *DPAGT1* groups. Patients with these subtypes had mean T1w scores almost 3 times that of the control group. Despite the small group numbers, this is reflected in statistical analysis, which found the mean T1w score of the *GFPT1* and *DPAGT1* subtypes to be significantly different from that of the control population (*GFPT1, P* < 0.05; *DPAGT1, P* < 0.05). By contrast, the mean T1w scores of the AChR‐deficiency group (0.80), the *COLQ* group (0.55), and the subject with *CHAT* mutations (0.53) were not significantly different from controls. However, 6 of 8 subjects with the latter 3 subtypes were the youngest in the CMS cohort. Example T1w images are shown in Figure 2.

### STIR Sequences

STIR sequence findings are shown for individual CMS subjects (see Table S1 in Supplementary Material online) and control subjects (Table 4), according to the categories defined in the Methods. Generally, STIR imaging did not show any overt differences between the CMS and control groups and was completely normal in all thigh muscles. Only 1 subject (subject 22, Table S1), with *GFPT1* CMS, had marked STIR hyperintensity in medial gastrocnemius bilaterally (Fig. 2H).

Medial gastrocnemius was the most common muscle in which mild hyperintensity was detected, affecting 13 CMS subjects (7 bilaterally) and 6 controls (all bilaterally). One difference between CMS and control groups was that STIR hyperintensity only affected the distal portion of medial gastrocnemius in controls, but it was also seen proximally in 8 of 13 CMS subjects. One patient with *DOK7* CMS (subject 12, Table S1) exhibited extensive mild STIR hyperintensity in both calves, with 12 of 14 muscles showing abnormality. No other subject had more than 6 muscles with mild hyperintensity, and in the majority only 1 or 2 muscles were affected. In 2 CMS subjects (subjects 3 and 7, Table S1) and faintly in 2 control subjects, a STIR hyperintense “central stripe” was noted, corresponding to the muscle end‐plate region of medial gastrocnemius (Fig. 2I). In subject 3 this feature was also seen in soleus bilaterally.

### Correlation with Clinical Parameters

**Figure 3:**
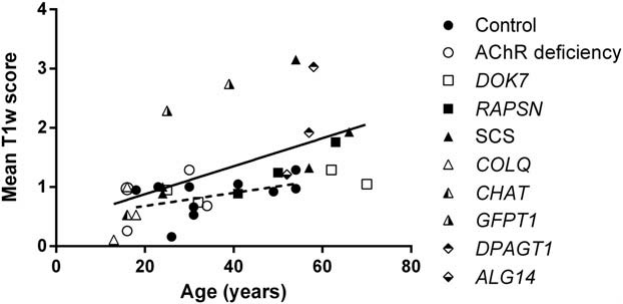
Correlation of mean T1w score and age. A statistically significant correlation is seen in the congenital myasthenic syndrome group (solid line: Spearman rho = 0.68, *P* < 0.0005), but not in the control group (dashed line: Spearman rho = 0.31, *P* = NS).

Mean T1w score correlated with increasing age in the CMS group as a whole (Fig. 3). All CMS subjects < 20 years of age had either AChR deficiency, *CHAT*, or *COLQ* subtypes. No significant correlation with age was seen in the control group. Correlation between the QMG and MGADL scores was evident (rho = 0.59, *P* < 0.005).

No statistically significant correlation was evident between mean T1w score and any of the overall clinical parameters. When either thigh‐ or calf‐level mean T1w score of only those subtypes that had abnormal T1w imaging (i.e., showed mild, extensive, or marked changes by severity categorization, in *RAPSN, DOK7*, SCS, *GFPT1*, and *DPAGT1*) were correlated with the relevant clinical outcomes, then a statistically significant inverse correlation was seen with ankle dorsiflexion MRC score only (rho = –0.55, *P* < 0.05, data not shown). Of note, there were no significant correlations between the pairs: (a) knee extension strength and quadriceps T1w muscle scores and (b) knee flexion strength and hamstrings T1w scores, and there was only a trend (rho = –0.5, *P* = 0.058) for the ankle dorsiflexion strength correlation with anterior compartment T1w scores.

### Imaging of a Patient with *ALG2* CMS

The 3‐T MRI scans from a patient with *ALG2* CMS were also available for review. This is another rare form of CMS caused by a glycosylation pathway gene mutation. These scans were performed at a different center using an MRI protocol that resulted in images of lower resolution. The patient was a 61‐year‐old man with onset of waddling gait and falls at age 4. Tubular aggregates were evident on muscle biopsy at 50 years of age. The patient showed marked proximal limb weakness, although he could still walk 15–20 meters unaided. A recent QMG score was 13 (of 39), and his condition has been responsive to pyridostigmine.

MRI at age 60 showed moderate atrophy of vastus lateralis (Fig. 1J). There was evidence of fatty infiltration in thigh and calf muscles with an overall mean T1w score of 1.42. However, there was marked disparity of involvement between thigh and calf muscles. The thigh muscles were severely affected (categorized as marked T1w severity) with a mean thigh T1w score of 2.08. Muscles of the posterior compartment of the thigh were most severely affected. By contrast, there was minimal involvement of calf muscles (mild limited severity and mean calf T1w score of 0.29). This degree of disparity was greater than that observed in any study subject. STIR imaging was not performed.

## DISCUSSION

We have shown that some CMS patients with *RAPSN, DOK7*, SCS, *GFPT1*, and *DPAGT1* mutations have T1w abnormalities on muscle MRI, and the most predictable abnormalities were seen in those with *GFPT1* and *DPAGT1* glycosylation pathway mutations. The main differences were seen on T1w imaging, which detects fatty infiltration of muscles, suggesting progressive muscle damage. A non‐selective pattern of muscle involvement was observed, which sets MRI findings in CMS apart from the highly selective patterns typical of many myopathic and dystrophic disorders.

Scans from 4 of the 5 subjects with glycosylation pathway mutations (with *GFPT1* or *DPAGT1* CMS) had the most severe fatty infiltration of our entire CMS cohort. Thigh‐ and calf‐level images from these subjects were unequivocally abnormal even in the youngest patient of this group, who was 25 years old. In both *GFPT1* and *DPAGT1* subtypes the myasthenic disorder is thought to arise from aberrant glycosylation of synaptic components. Glycosylation is a ubiquitous posttranslational modification known to be biologically significant in muscle, with >200 known glycosyltransferases residing in the Golgi apparatus.^33^ Therefore, it is possible mutations in these genes could have additional direct pathological effects in extra‐synaptic regions. Muscle specimens of patients with *DPAGT1* myasthenia have shown prominent myopathic features, attributed to hypoglycosylation of multiple muscle fiber proteins, including fiber‐type disproportion, degenerating mitochondria, and destruction of the muscle fiber organelles associated with autophagy.^34^ Furthermore, unlike most other CMS subtypes, the *DPAGT1* and *GFPT1* myasthenia subtypes are associated with the appearance of tubular aggregates on muscle biopsy, and these inclusions were detected on muscle biopsy of all 4 patients with these subtypes in our study. By contrast, MRI findings from the subject with *ALG14* CMS, also caused by a glycosylation pathway gene mutation, were not nearly as severe. This was despite disease duration of 47 years and clinical features indicative of significant weakness. This patient has not undergone muscle biopsy, although her similarly affected sibling has had 2 biopsies, neither of which has demonstrated tubular aggregates. The images from the patient with *ALG2* CMS also showed marked fatty infiltration, predominantly affecting posterior thigh muscles. Although this patient's imaging was not acquired within our main study protocol, the findings are noteworthy, as this subtype is also caused by glycosylation pathway abnormality and is associated with tubular aggregates, which were present in this patient.

No difference from control group T1w imaging was apparent in the 4 subjects with AChR deficiency or in the subjects with *COLQ* and *CHAT* CMS. Despite their essentially normal MRI scans the patients with AChR deficiency were among the weakest in the entire cohort. It may be significant that this group was relatively young compared with the other subgroups. Similarly, the 3 patients with *COLQ* mutations were among the youngest in the study, with a mean age of just 15.7 years. Further imaging of older patients with AChR deficiency, *CHAT*, and *COLQ* CMS is desirable to investigate whether fatty infiltration may develop over a longer period of time in these subtypes. Likewise, imaging of younger patients with other subtypes, to investigate whether MRI abnormality is present in childhood, is also desirable. In the remainder of the subtypes, SCS, *RAPSN*, and *DOK7*, the findings were mixed. Some subjects fell into the categories of mild, extensive, or marked T1w fatty infiltration, and the others fell into the normal or mild limited categories that encompassed all healthy controls. The correlation of mean T1w score and age would suggest that fatty infiltration increases over time and is related to disease duration.

Unsurprisingly, the clinical scores that capture weakness outside of the lower extremities did not correlate with mean lower limb muscle T1w scores. The lack of correlation between the MRI T1w scores and the relevant muscle strength scores in those CMS subtypes affecting muscle MRI could be due to a number of factors, including: (a) the neuromuscular transmission defect may be contributing to the muscle weakness; and (b) the MRC score range was a narrow and insensitive score (most weak muscles score 4 or 4^+^ despite covering a wide range of strengths). The correlation between ankle dorsiflexion and the anterior lower leg compartment approached significance.

STIR hyperintensity results from prolongation of T2 relaxation times and is typically attributed to muscle edema or inflammation, although the underlying molecular basis is poorly understood.^11^ Only minor differences in STIR imaging between the CMS and control cohorts were observed. Marked STIR hyperintensity was seen in just 1 subject who has *GFPT1* CMS and had markedly abnormal T1w imaging. When mild STIR hyperintensity of medial gastrocnemius was detected, it was limited to the distal portion of the muscle in all control subjects but in just over half of the CMS subjects. Therefore, proximal STIR hyperintensity of the gastrocnemius may be more likely to be associated with pathology than limited distal involvement, although this finding is fairly subtle.

A notable finding is that some T1w hyperintensity within muscles, indicating fatty infiltration, was seen in all healthy controls, and over half had mild STIR hyperintensity in predominantly distal gastrocnemius. Semiquantitative MRI analysis of skeletal muscle in healthy subjects is rare, and just 2 studies employing similar methods were identified.^31^, ^35^ Both studies, 1 including adults and the other children, demonstrated mild fatty infiltration in healthy controls on T1w imaging, with the former study also reporting mild STIR hyperintensity, again predominantly in distal gastrocnemius muscles. This underscores the importance of establishing normal control appearances before assessing mild MRI hyperintensity as an abnormality. These should be acquired on the same MRI scanner, as field inhomogeneities are scanner‐specific.

As with most other published studies of muscle MRI findings in rare inherited neuromuscular disorders, this study is limited by small patient numbers, variation in ages, and disease duration between subjects. It is unlikely that these factors can be fully eliminated, mainly because the congenital myasthenic syndromes are collectively and individually particularly rare. These limitations aside, our study benefited from access to a wide range of CMS patients, covering all main phenotypes, which allowed us to cross‐compare with each other and with the control group, thereby eliminating normal changes.

Further imaging should help to expand our appreciation of the range of findings in this group of disorders. It would be worthwhile to image both a greater number of patients and to specifically image patients with less common phenotypes, such as the recently reported distal variant caused by mutations in *AGRN*.^36^ In addition, imaging different body regions or whole‐body muscle MRI^8^ may detect muscle MRI changes in more CMS genotypes. For example, in SCS, predominant weakness of the finger extensors^20^, ^37^ and thumb abductors^38^ is well documented. It would be of interest to see if MRI abnormalities exist in these affected muscles (note that 3 of 5 SCS patients had calf muscle MRI T1w abnormalities, including 1 with distal weakness).

In conclusion, this study has shown that MRI in CMS muscle is likely to show either a non‐selective distribution of fat infiltration or a scan within normal limits in the setting of significant clinical weakness. This non‐selective muscle involvement is in contrast to the highly selective patterns typical of many myopathic and dystrophic disorders. Muscle MRI could play an adjunctive role in investigation of CMS, specifically in differentiating CMS from myopathic and dystrophic disorders and between CMS subtypes. The lack of an association between muscle strength and muscle MRI highlights the usefulness of this technique in providing additional information beyond clinical features. This suggests muscle MRI can be a non‐invasive tool to detect muscle involvement in those subtypes of CMS where myopathic changes have been described previously. Measures of T2, such as STIR imaging, are less likely to be useful than T1w imaging, as abnormality was rarely observed.

The authors are grateful to the patients who participated in the study, and their families. We also thank Jane Francis (chief MR technologist) and Dr. Malenka Bissell of the University of Oxford Centre for Clinical Magnetic Resonance Research, John Radcliffe Hospital, Oxford, UK, for their help and advice in performing scans. We also thank Professor Francesco Muntoni of the Dubowitz Neuromuscular Centre, Institute of Child Health, University College London, UK, and Prof. Peter Jezzard, University of Oxford Centre for Clinical Magnetic Resonance Research, for helpful discussion and guidance.

## Supporting information

Additional supporting information may be found in the online version of this article

Supporting Information Table 1.
