# Supplementary material for: Muscle magnetic resonance imaging in congenital myasthenic syndromes
Source: Muscle Nerve. 2016 Feb 22;54(2):211–9. doi: 10.1002/mus.25035 (PMC4982021; doi:10.1002/mus.25035)
Supplement: Supplementary file 1 — Supporting Information Table 1. [file MUS-54-211-s001.docx]

**Supplementary Table 1. Clinical and imaging findings in CMS subjects.**

| **Subject ID** | **Gender/current age (years)** | **MGADL score** | **QMG score** | **Timed leg raise (s)** | **Timed 10 metre walk (s)** | **Thigh** | | **Calf** | | | **Mean T1w score** |
| --- | --- | --- | --- | --- | --- | --- | --- | --- | --- | --- | --- |
|  |  |  |  |  |  | **T1w** | **STIR** | **T1w** | **STIR** | |  |
| **AChR def** |  |  |  |  |  |  |  |  |  | |  |
| 1 | W/16 | 6 | 13 | 38 | 5.9 | - | - | - | - | | 0.26 |
| 2 | W/30 | 9 | 16 | 3.5 | unable | +/- | - | +/- | +/- | | 1.29 |
| 3 | M/34 | 9 | 17 | 19.5 | unable | +/- | - | +/- | - | | 0.68 |
| 4 | M/16 | 9 | 24 | 0 | unable | +/- | - | +/- | - | | 0.97 |
| **mean** | **24** | **8.3** | **17.5** | **15.3** |  |  |  |  |  | | **0.80** |
| ***RAPSN*** |  |  |  |  |  |  |  |  |  | |  |
| 5 | M/63 | 2 | 14 | 19 | 7.5 | ++ | - | + | - | | 1.76 |
| 6 | W/41 | 5 | 6 | 56 | 6.6 | - | - | +/- | +/- | | 0.89 |
| 7 | M/50 | 4 | 7 | 49 | 6.8 | +/- | - | ++ | +/- | | 1.24 |
| **mean** | **51.3** | **3.7** | **9** | **41.3** | **7.0** |  |  |  |  | | **1.30** |
| ***DOK7*** |  |  |  |  |  |  |  |  |  | |  |
| 8 | M/40 | 10 | 16 | 87.5 | 7.6 | + | - | + | +/- | | 1.74 |
| 9 | M/25 | 0 | 3 | 100 | 6 | - | - | +/- | - | | 0.95 |
| 10 | W/62 | 5 | 16 | 24.5 | 11.5 | +/- | - | nd | nd | | 1.29 |
| 11 | W/32 | 11 | 16 | 19.5 | 8.3 | - | - | +/- | +/- | | 0.74 |
| 12 | W/70 | 7 | 15 | 25 | 15 | +/- | - | +/- | + | | 1.05 |
| **mean** | **45.8** | **6.6** | **13.2** | **51.3** | **9.7** |  |  |  |  | | **1.15** |
| **SCS** |  |  |  |  |  |  |  |  |  | |  |
| 13 | W/54 | 8 | 7 | 37 | 12.3 | ++ | - | ++ | +/- | | 3.15 |
| **Subject ID** | **Gender/current age (years)** | **MGADL score** | **QMG score** | **Timed leg raise (s)** | **Timed 10 metre walk (s)** | **Thigh** | | **Calf** | | | **Mean T1w score** |
|  |  |  |  |  |  | **T1w** | **STIR** | **T1w** | | **STIR** |  |
| 14 | M/57 | 8 | 22 | 85.5 | 7.2 | +/- | - | + | +/- | | 1.32 |
| 15 | M/66 | 3 | 14 | 29 | 7.6 | nd | nd | ++ | + | | 1.93 |
| 16 | M/24 | 3 | 3 | 100 | 4.9 | - | - | +/- | +/- | | 1.0 |
| 17 | W/24 | 9 | 16 | 29 | 6.2 | +/- | - | +/- | +/- | | 0.89 |
| **mean** | **45** | **6.2** | **12.4** | **56.1** | **7.7** |  |  |  |  | | **1.66** |
| ***COLQ*** |  |  |  |  |  |  |  |  |  | |  |
| 18 | W/16 | 1 | 7 | 41 | 8.5 | +/- | - | +/- | - | | 1.0 |
| 19 | W/13 | 6 | 19 | 8 | 6.1 | - | - | - | - | | 0.11 |
| 20 | W/18 | 6 | 18 | 13 | 6.5 | - | - | - | - | | 0.53 |
| **mean** | **15.7** | **4.3** | **14.7** | **20.7** | **7.0** |  |  |  |  | | **0.55** |
| ***CHAT*** |  |  |  |  |  |  |  |  |  | |  |
| 21 | M/16 | 6 | 13 | 3 | 5.7 | - | - | +/- | +/- | | 0.53 |
| ***GFPT1*** |  |  |  |  |  |  |  |  |  | |  |
| 22 | M/39 | 3 | 7 | 20 | 6.9 | ++ | - | ++ | ++ | | 2.74 |
| 23 | M/25 | 2 | 13 | 12.5 | 9.4 | ++ | - | ++ | +/- | | 2.29 |
| **mean** | **32** | **2.5** | **10** | **16.3** | **8.2** |  |  |  |  | | **2.51** |
| ***DPAGT1*** |  |  |  |  |  |  |  |  |  | |  |
| 24 | W/57 | 8 | 18 | 33.5 | nd | ++ | - | + | +/- | | 1.92 |
| 25 | W/58 | 3 | 18 | 0 | unable | ++ | - | ++ | + | | 3.03 |
| **mean** | **57.5** | **5.5** | **18** | **16.8** |  |  |  |  |  | | **2.47** |
| ***ALG14*** |  |  |  |  |  |  |  |  |  | |  |
| 26 | W/52 | 5 | 15 | 9 | 7.6 | +/- | - | +/- | - | | 1.21 |

AChR def**;** acetylcholine receptor deficiency syndrome**,** SCS; slow channel syndrome, W; woman, M; man, QMG; quantitative myasthenia gravis, MGADL; myasthenia gravis activities of daily living, s; seconds, T1w; T1-weighted, STIR; short-tau-inversion-recovery, nd; not done, nd; -; normal, +/-; mild limited changes, +; mild extensive changes, ++; marked changes (as defined in the methods).
